# Diabetic and Elder Patients Experience Superior Cardiovascular Benefits After Gastric Bypass Induced Weight Loss

**DOI:** 10.3389/fendo.2018.00718

**Published:** 2018-11-28

**Authors:** Pedro R. Pereira, Marta Guimarães, Tiago Morais, Sofia S. Pereira, Mário Nora, Mariana P. Monteiro

**Affiliations:** ^1^Endocrine, Cardiovascular and Metabolic Research, Unit for Multidisciplinary Research in Biomedicine, University of Porto, Porto, Portugal; ^2^Department of Anatomy, Abel Salazar Institute of Biomedical Sciences, University of Porto, Porto, Portugal; ^3^Department of General Surgery, Centro Hospitalar de Entre o Douro e Vouga, Santa Maria da Feira, Portugal

**Keywords:** cardiovascular risk, cardiovascular outcome, obesity, bariatric/metabolic surgery, diabetes

## Abstract

**Background/Objetives:** Obesity and obesity related co-morbidities are well-recognized risks for cardiovascular (CV) disease and mortality. Weight loss improves CV risk factors and the efficacy of bariatric surgery in decreasing CV mortality is now well-established. Our aim was to assess CV risk progression and occurrence of CV events in a cohort of patients that underwent Roux-en-Y gastric bypass (RYGB) for obesity treatment in a single academic public center.

**Subjects and Methods:** Ten year CV risk was estimated using the Framingham Equation at baseline and 2 years after RYGB surgery in our patients cohort (*n* = 260). In the subgroup with a follow-up time longer than 4 years after surgery (*n* = 185; mean 5.4 ± 0.1 years), CV risk adjusted for the time length after RYGB was similarly estimated and the occurrence of CV events for outcome adjudication was monitored during the same time period by reviewing the hospital patients' record, the electronic national health system patient register and our center outpatient clinic records.

**Results:** Ten year CV risk was significantly reduced 2 years after surgery when compared to baseline, with reductions of 1.65 ± 0.25% in the risk of CV disease. Patients with prior type 2 diabetes and aged 50 years or older experienced a significantly superior CV risk reduction, with diabetic patients experiencing a reduction of their 10–year CV disease risk of 3.58 ± 1.11% vs. a reduction of 1.31 ± 0.20% in non-diabetic patients and with the 10–year risk of CV disease dropping 3.41 ± 0.75% in patients older than 50 vs. a reduction of 0.99 ± 0.18 in patients up to 50 years. For the subgroup of patients with a longer follow-up time, baseline CV risk estimation predicted the occurrence of 6.08 ± 0.56 cardiovascular disease (CVD) events, 3.87 ± 0.39 coronary heart disease (CHD) events, 1.49 ± 0.22 myocardial infarctions (MI), 0.71 ± 0.09 strokes, 0. 28 ± 0.05 deaths from CHD and 0.35 ± 0.05 deaths from CVD. No CV events were adjudicated in this subgroup during follow-up.

**Conclusions:** RYGB significantly improves CV risk and prevents the occurrence of CV events. For similar weight loss, diabetic and elder patients experience a superior CV risk improvement and may have additional CV benefits after bariatric surgery.

## Introduction

Obesity and obesity-related morbidities are well-established risks for cardiovascular (CV) disease and mortality (1). Obesity has direct effects on CV structure by increasing myocardial fat deposition and altering hemodynamic function (2). In addition, obesity triggers an increased sympathetic activity and a decreased systemic vascular resistance, which result in increased heart rate and cardiac output, generating cardiac overload and eventually leading to left ventricular hypertrophy, atria dilation (3), heart dysfunction and dysrhythmia (4). Moreover, obesity is the central feature of the metabolic syndrome, which includes other CV risk factors such as high blood pressure (BP), lipid abnormalities and altered glucose homeostasis or type 2 diabetes (T2D) (5). Indeed, obese individuals were shown to have at least twice the risk of developing heart failure compared to normal weight individuals (2, 6). Therefore, obesity is undeniably an independent predictor of CV disease (2, 6).

Weight loss allows the improvement of several CV risk factors and was demonstrated not only to decrease overall mortality, but also to specifically prevent CV related mortality (7–9). Bariatric surgery is the most effective means to achieve substantial and sustained weight loss in severely obese patients in whom previous conservative or medical interventions have repeatedly failed (10). Furthermore, there is now considerable evidence derived from prospective trials and meta-analyses that demonstrate the benefits of weight loss attained through bariatric surgery in improving CV risk factors, including reducing BP (11), improving dyslipidemia (12) and ameliorating glycemic control or even inducing clinical remission of T2D (13–15). Moreover, bariatric surgery not only proved to reduce CV risk scores, but also prevents the occurrence of CV events and reduces overall mortality (7, 16). In the Swedish Obesity Subjects Study (SOS study), post-bariatric patients when compared to non-operated weight matched controls, experienced 33% and 53% fewer total and fatal CV events, respectively (17).

Our aim was to evaluate the impact of weight loss attained through Roux-en-Y gastric bypass (RYGB) on CV outcomes by comparing the predicted vs. occurred CV event rate in a cohort of post-RYGB patients attended at a single academic public center.

## Methods

### Subject selection and enrolment criteria

Subjects enrolled in this study were attended at a single academic public center for Obesity Treatment. To be considered candidates for bariatric surgery patients had to meet the criteria of having a body mass index (BMI) over 40 kg/m^2^ or over 35 kg/m^2^ in the presence of obesity-related morbidities, provided previous non-invasive weight loss interventions had failed and there were no contraindications for the proposed surgical intervention. Participants were selected from our prospective database of post bariatric patients operated between 2005 and 2010 (*N* = 917) based on the following criteria: (1) availability of all clinical and analytical parameters required for CV risk calculation (*N* = 315) using the Framingham Heart Study (2008) score (as detailed in the section below), (2) having a minimum follow-up time of 2 years after surgery and (3) having a least of one observation in our center per year during the time of follow-up (*N* = 55 lost to follow-up), yielding the final subset of patients for data analysis (*n* = 260). To ensure homogeneity of clinical data acquisition and recording in our post-bariatric prospective register, all patients were attended by the same multidisciplinary team that includes endocrinologists, surgeons, psychologists, and nutritionists before and after the RYGB surgery.

### Surgical procedure

The RYGB procedure consisted in the creation of a neo-stomach with an approximate volume of 30 cm^3^, which was anastomosed to a jejunum loop located 50–60 cm from the ligament of Treitz (gastro-jejunostomy), while the biliopancreatic flow was restored by an entero-enteric anastomosis performed 90 to 120 cm distal to the gastro-jejunostomy. Thereby, RYGB excludes from the gastrointestinal transit approximately 95% of the stomach, the entire duodenum and part of the jejunum.

### CV risk calculation

To estimate CV risk (coronary, cerebrovascular, peripheral arterial disease and heart failure), the updated sex-specific multivariable risk factor algorithm derived from the Framingham Heart Study (2008) was used. This algorithm estimates an individual's overall risk for cardiovascular disease (CVD), as well as the isolated risk for coronary heart disease (CHD), myocardial infarction (MI), stroke, peripheral vascular disease, death from CHD, death from CVD and heart failure in a given period of time spanning from 4 to 10 years. To estimate the risk for each CV event, the Framingham equation uses fasting blood glucose, diagnosis of diabetes, blood pressure, total cholesterol, HDL cholesterol, presence of left ventricular hypertrophy (LVH), tobacco use and family history of CV events (18). Of note that the Framingham algorithm does not specifically include body weight nor BMI for CV risk calculation.

CV risk was estimated before (baseline T0) and 2 years after surgery (T2) in the entire patient cohort. In the subgroup with a follow-up time longer than 4 years after surgery (*n* = 185; mean 5.4 ± 0.01 years), CV risk adjusted for the time length after RYGB was similarly estimated. Occurrence of CV events was correspondingly monitored in this patient subgroup and for the same time period. The means used for CV event adjudication included: (1) review of hospital patient records for the occurrence of CV events requiring in-hospital admission, (2) review of electronic national health system patient register depicting outpatient clinical records during routine appointments at the general practitioner and (3) review of our center outpatient clinic records. The protocol was reviewed and approved by the Institutional Ethical Review and Hospital Administration Boards in accordance with the recommendations of the Declaration of Helsinki and the European Data Protection Regulations.

### Statistical analysis

Results are presented as mean ± standard error of the mean (Mean ± SEM) unless otherwise specified. D'Agostino & Pearson omnibus test was used to determine the normality of the groups. Comparisons between three or more groups were performed with the Kruskal–Wallis test followed by the Dunn *post-hoc* test or with One-way ANOVA followed by a Holm-Sidak *post-hoc* test accordingly to the normality of the data. The difference between two independent experimental groups was evaluated using the unpaired Student *t*-test or Mann-Whitney U accordingly to the normality of the data. A *p* < 0.05 was considered statistically significant. All statistical analyzes were performed with the aid of the GraphPad Prism software version 7.00 for windows.

## Results

This post-RYGB patient cohort (*n* = 260) encompassed 91.6% females (*n* = 238) and 8.4% males (*n* = 22). At the time of surgery, subjects had a mean age of 42.12 ± 0.62 years and a mean BMI of 43.9 ± 0.4 kg/m^2^, while 14.9% (*n* = 39) had been diagnosed with type 2 diabetes (T2D) prior undergoing bariatric surgery. Baseline patient demographics, anthropometric and biochemical features, irrespectively of specific drug treatment for any of these conditions are depicted on Table 1.

**Table 1:** Baseline patient demographics, anthropometric and biochemical features.

Age (years)	42.12 ± 0.62
Male:Female	22:238 91.6%:8.4%
BMI (Kg/m^2^)	43.9 ± 0.4
Fasting glucose (mg/dL)	107 ± 2
HbA1c (%)^(1)^	5.0 ± 0.1
Systolic BP (mm Hg)	137 ± 1
Diastolic BP (mm Hg)	81 ± 1
Total Cholesterol (mg/dL)	195 ± 2
Triglycerides (mg/dL)	133 ± 4
HDL (mg/dL)	46 ± 1
LDL (mg/dL)	122 ± 2
Type 2 Diabetes (*n*, % of patients)	39 (14.9%)
Anti-diabetic drug (*n*, % of patients)	39 (14.9%)
Anti-hypertensive drug (*n*, % of patients)	63 (24.1%)
Anti-dyslipidemia drug (*n*, % of patients)	36 (13.8%)

(1) *HbA1c (%) value is for patient cohort (n = 260)*.

Patients had an average time after RYGB surgery of 4.41 ± 0.11 years, spanning from a minimum of 2 years to a maximum of 7 years, with the subsequent follow-up time distribution: 4 years 20.7% (*n* = 54), 5 years 17.3% (*n* = 44), 6 years 16.9% (*n* = 43) and 7 years 17.3% (*n* = 44). A maximum percentage of excess BMI loss [%EBMIL = 100^*^(BMI_Baseline_-BMI_Follow−up_)/(BMI_Baseline_-25)] of 76.84% ± 1.33% was achieved 2 years after surgery (Table 2). After RYGB surgery there was a significant improvement of several independent CV risk factors, including a decrease in BMI, fasting glucose, total cholesterol, LDL cholesterol, triglycerides, systolic, and diastolic blood pressure, along with an increase in HDL cholesterol (Table 3).

**Table 2:** Patient population distribution according to follow-up time and corresponding BMI and EBMIL.

**Follow-up Time**	**N (%)**	BMI (Kg/m^2^)^*^	EBMIL (%)^**^
*2 Years*	260 (100)	29.91 ± 0.29	76.84 ± 1.33
*4 Years*	54 (20.8)	31.02 ± 0.65	71.31 ± 2.42
*5 Years*	44 (16.9)	30.97 ± 0.80	71.52 ± 3.30
*6 Years*	43 (16.6)	33.02 ± 0.68	54.49 ± 4.22
*7 Years*	44 (16.9)	31.72 ± 0.68	65.70 ± 3.18

* BMI, body mass index;

** *EBMIL, excess BMI loss*.

**Table 3:** Patient anthropometric and biochemical features change from baseline at 2 years of follow-up.

	**Baseline**	**2 y after surgery**	**Δ**	***p***
BMI (Kg/m^2^)	43.90 ± 0.39	29.92 ± 0.28	−13.98 ± 0.27	< 0.001
Fasting glucose (mg/dL)	107 ± 2	87 ± 1	−19 ± 2	< 0.001
Total Cholesterol (mg/dL)	195 ± 2	176 ± 2	−19 ± 2	< 0.001
Triglycerides (mg/dL)	133 ± 4	84 ± 2	−49 ± 4	< 0.001
LDL (mg/dL)	122 ± 2	102 ± 1	−20 ± 2	< 0.001
HDL (mg/dL)	46 ± 1	57 ± 1	11 ± 1	< 0.001
Systolic BP (mm Hg)	137 ± 1	134 ± 1	−3 ± 1	0.03
Diastolic BP (mm Hg)	81 ± 1	79 ± 1	−2 ± 1	0.04

**Figure 1:**
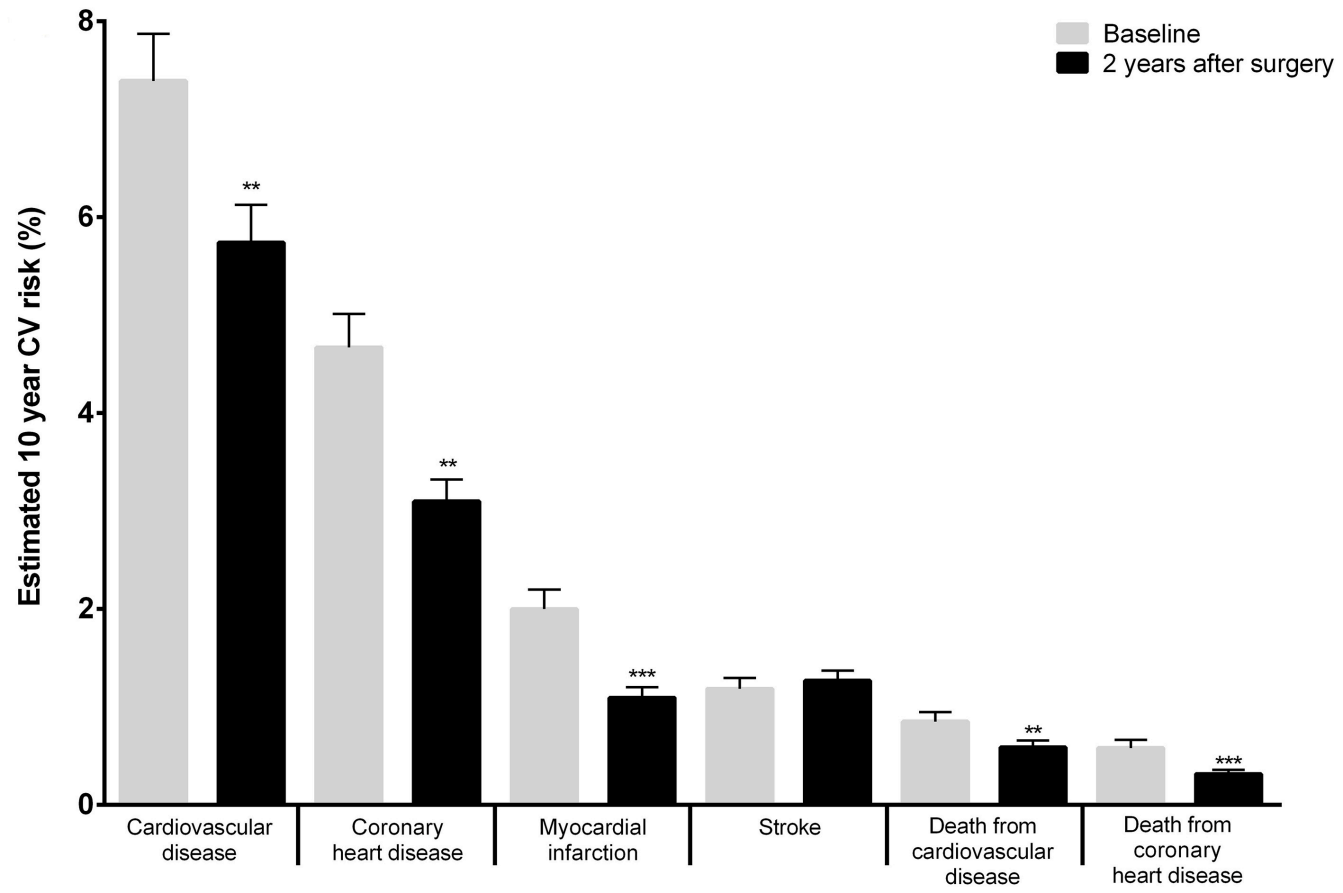
Estimation of the 10-year risk of CV events applying the Framingham Heart Study (2008) score to patients' clinical and analytical features at baseline and 2 years after surgery. As an example, the Framingham Cardiovascular Risk equation predicted a baseline (pre-operative) 10-year risk for CVD events of 7.39 ± 0.49% and 2 years after surgery this risk was of 5.74 ± 0.38% (**p < 0.01; ***p <0.001–baseline vs. 2 years after surgery).

At baseline, the 10-year CV risk predicted by the Framingham Cardiovascular Risk Equation was 7.39 ± 0.48% for CVD, 4.67 ± 0.34% for CHD, 1.99 ± 0.20% for MI, 1.18 ± 0.11% for stroke, 0.58 ± 0.08% for death from CHD and 0.84 ± 0.10% for death from CVD (Figure 1). The 10-year CV risk recalculated 2 years after surgery decreased significantly predicting a risk of 5.74 ± 0.38% for CVD, 3.1 ± 0.22% for CHD, 1.09 ± 0.11% for MI, 1.27 ± 0.10% for stroke, 0.31 ± 0.04% for death from CHD and 0.59 ± 0.07% for death from CVD (Figure 1). Two years after RYGB surgery the 10-year CV risk reduction was statistically significant for most isolated CV events (−1.65 ± 0.25% for CVD, −1.57 ± 0.20% for CHD, −0.91 ± 0.13% for MI, −0.27 ± 0.06% for death from CHD and −0.26 ± 0.06% for death from CVD), with the sole exception of the risk for stroke that did not vary considerably since baseline (0.08 ± 0.08% for stroke; Figure 1).

**Figure 2:**
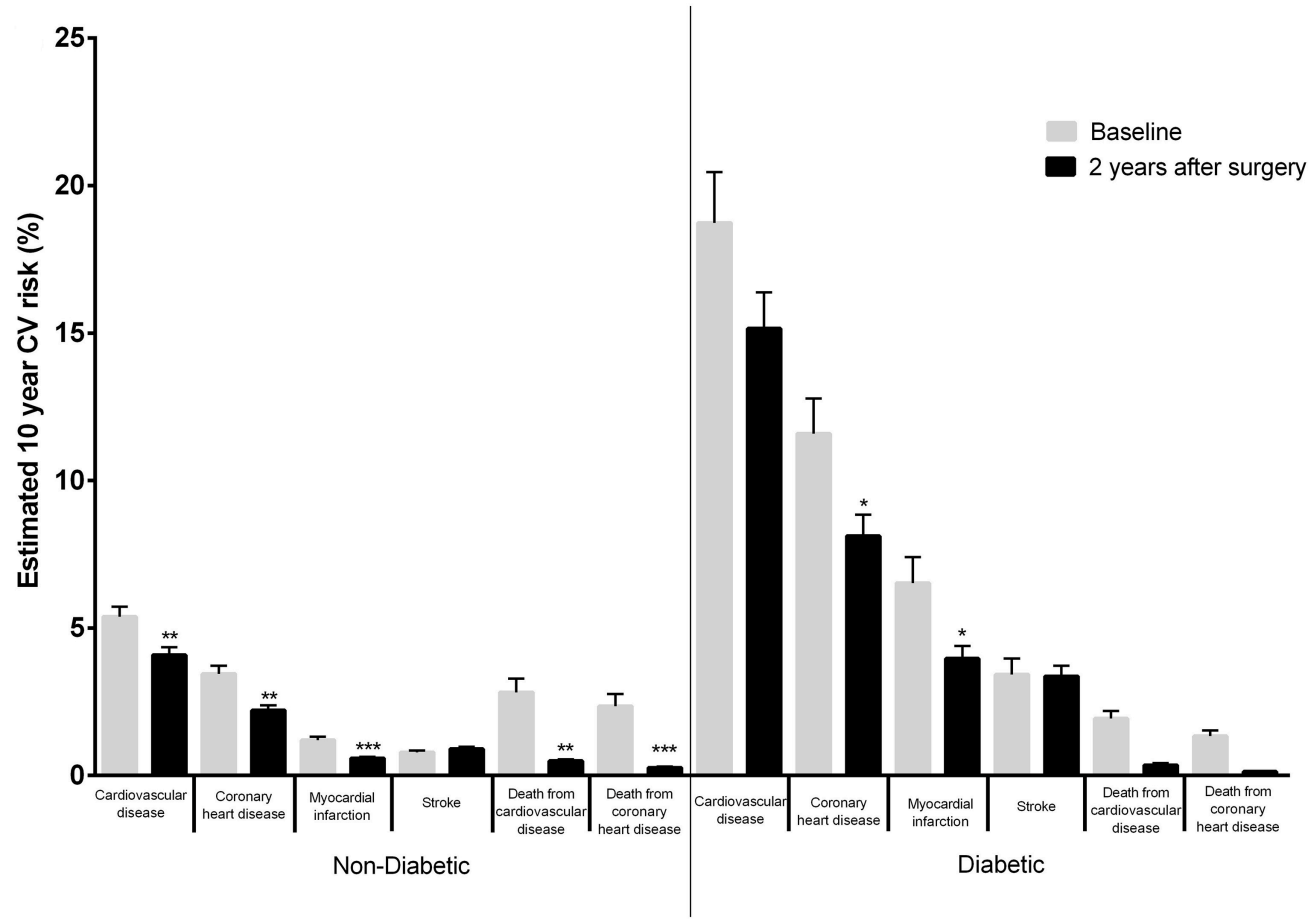
Estimated 10-year CV risk applying the Framingham Heart Study (2008) score to patients' clinical and analytical data at baseline and 2 years after surgery in diabetic and non-diabetic patients. As an example, in non-diabetic patients, the Framingham Cardiovascular Risk equation predicted a pre-surgical 10-year risk of CVD events of 5.39 ± 0.34 and a 10-year risk for CVD events 2 years after surgery of 4.08 ± 0.28% while in diabetic patients, the Framingham Cardiovascular Risk equation predicted a pre-surgical 10-year risk of CVD events of 18.74 ± 1.72% and a 10-year risk for CV events 2 years after surgery of 15.16 ± 1.22% for CVD (*p < 0.05; **p < 0.01; ***p < 0.001–baseline vs. 2 years after surgery).

**Figure 3:**
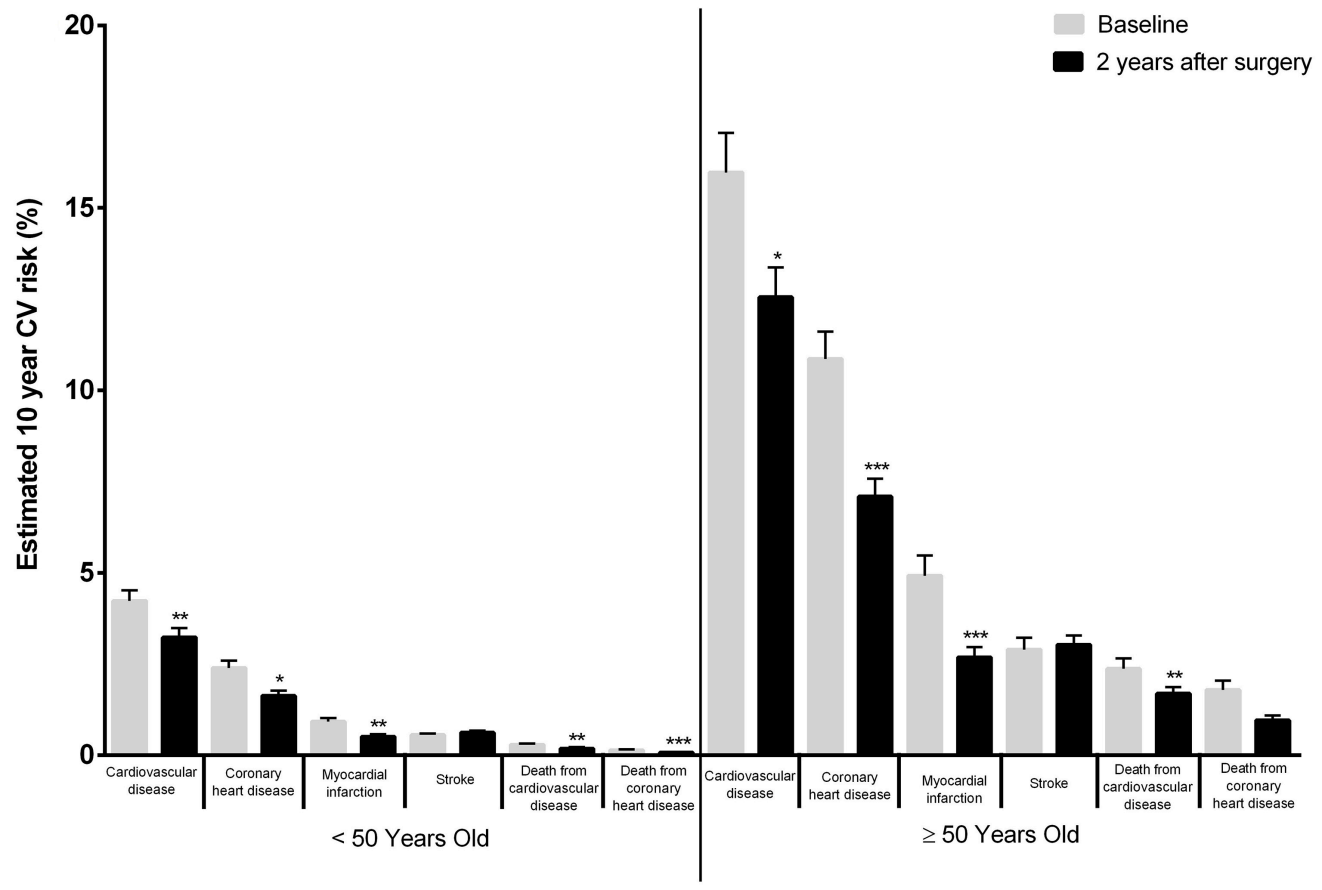
Estimated 10-year CV risk in different age groups applying the Framingham Heart Study (2008) score to patients' clinical and analytical data prior to surgery and 2 years after surgery. As an example, the Framingham Cardiovascular Risk equation predicted a pre-surgical 10-year risk of CVD events of 4.23 ± 0.29% in patients younger than 50 while in patients with at least 50 years the same risk was of 15.97 ± 1.09%. Two years after surgery the 10-year risk of CVD had drop to 3.23 ± 0.25% for patients younger than 50 while for patients with at least 50 years it had drop to 12.55 ± 0.82% (*p < 0.05; **p < 0.01; ***p < 0.001–baseline vs. 2 years after surgery).

Patients diagnosed with T2D prior to surgery had a significant decrease in 10-year risk for most CV events when compared to non-diabetic individuals, once excluded the risk for stroke (Figure 2). In addition, patient stratification according to age [ < 50 years (*n* = 182) and ≥ 50 years (*n* = 82)], showed that the elder group experienced a significant 10-year CV risk reduction when compared to the younger group (Figure 3); these differences prevailed even after excluding T2D patients from the analysis, as the condition was more frequent in the elder patient group (Supplementary Data, Figure S1, Table S1). Apart from fasting glucose levels that decreased significantly in T2D patients as compared to non-diabetic patients, the change from baseline of all other CV risk parameters that were evaluated experienced similar improvements, including BMI (Table 4). Similar findings were observed when the clinical profiles of younger and older patients before and after surgery were compared (Table 5).

**Table 4:** Comparison of clinical and biochemical features at baseline and 2 years after surgery according to the presence of type 2 diabetes prior to RYGB surgery.

	**Non-Diabetic**			**Diabetic**			**Change from baseline at 2 y**		
	**Baseline**	**2 y after surgery**	***p***	**Baseline**	**2 y after surgery**	***p***	**Non-Diabetic**	**Diabetic**	***p***
Age (years)	41.1 ± 0.7		48.2 ± 1.5						
Male/Female	18 8.1%		4 10.3%						
BMI (Kg/m2)	43.9 ± 0.4	29.8 ± 0.3	< 0.001	43.8 ± 1.4	30.8 ± 0.9	< 0.001	−14.2 ± 0.3	−12.9 ± 0.8	0.05
Fasting glucose (mg/dL)	102 ± 2	86 ± 1	< 0.001	134 ± 8	95 ± 3	< 0.001	−16 ± 2	−39 ± 7	< 0.001
Total Cholesterol (mg/dL)	195 ± 3	175 ± 2	< 0.001	195 ± 7	181 ± 6	0.10	−19 ± 3	−14 ± 6	0.31
Triglycerides (mg/dL)	130 ± 4	83 ± 2	< 0.001	148 ± 12	91 ± 5	< 0.001	−47 ± 4	−56 ± 10	0.31
LDL (mg/dL)	122 ± 2	102 ± 2	< 0.001	122 ± 5	106 ± 5	0.04	−21 ± 2	−15 ± 5	0.10
HDL (mg/dL)	46 ± 1	57 ± 1	< 0.001	44 ± 2	56 ± 2	< 0.001	11 ± 1	12 ± 2	0.65
Systolic BP (mm Hg)	137 ± 1	133 ± 1	0.03	141 ± 3	139 ± 3	0.64	−4 ± 1	−2 ± 4	0.66
Diastolic BP (mm Hg)	81 ± 1	79 ± 1	0.06	82 ± 2	81 ± 2	< 0.001	−2 ± 1	−1 ± 2	0.32

**Table 5:** Comparison of clinical and biochemical features at baseline and 2 years after surgery according to the patients age at the time of RYGB surgery.

	**Age under 50 y**			**Age over 50 y**			**Change from baseline at 2 y**		
	**Baseline**	**2 y after surgery**	***p***	**Baseline**	**2 y after surgery**	***p***	**Under 50 y**	**Over 50 y**	***p***
Age (years)	37.4 ± 0.5		55.1 ± 0.4						
Male/Female	15 7.9%		7 10.0%						
BMI (Kg/m2)	43.7 ± 0.4	29.7 ± 0.3	< 0.001	44.5 ± 0.9	30.5 ± 0.6	< 0.001	−14.0 ± 0.3	−14.1 ± 0.6	0.62
Type 2 diabetes (n. % of patients)	19 (10.0%)			20 (28.6%)					
Fasting glucose (mg/dL)	102 ± 2	86 ± 1	< 0.001	121 ± 5	91 ± 2	< 0.001	−16 ± 2	−30 ± 5	< 0.001
Total Cholesterol (mg/dL)	192 ± 3	174 ± 2	< 0.001	201 ± 4	181 ± 4	< 0.001	−18 ± 3	−20 ± 5	0.99
Triglycerides (mg/dL)	131 ± 5	82 ± 2	< 0.001	138 ± 7	90 ± 4	< 0.001	−49 ± 4	−47 ± 7	0.87
LDL (mg/dL)	120 ± 3	101 ± 2	< 0.001	128 ± 3	105 ± 3	< 0.001	−19 ± 2	−23 ± 4	0.86
HDL (mg/dL)	46 ± 1	56 ± 1	< 0.001	45 ± 1	58 ± 1	< 0.001	10 ± 1	13 ± 2	0.37
Systolic BP (mm Hg)	136 ± 1	131 ± 1	0.01	142 ± 2	141 ± 2	0.75	−4 ± 1	−1 ± 3	0.30
Diastolic BP (mm Hg)	80 ± 1	78 ± 1	0.06	82 ± 2	81 ± 2	0.40	−2 ± 1	−1 ± 2	0.95

**Figure 4:**
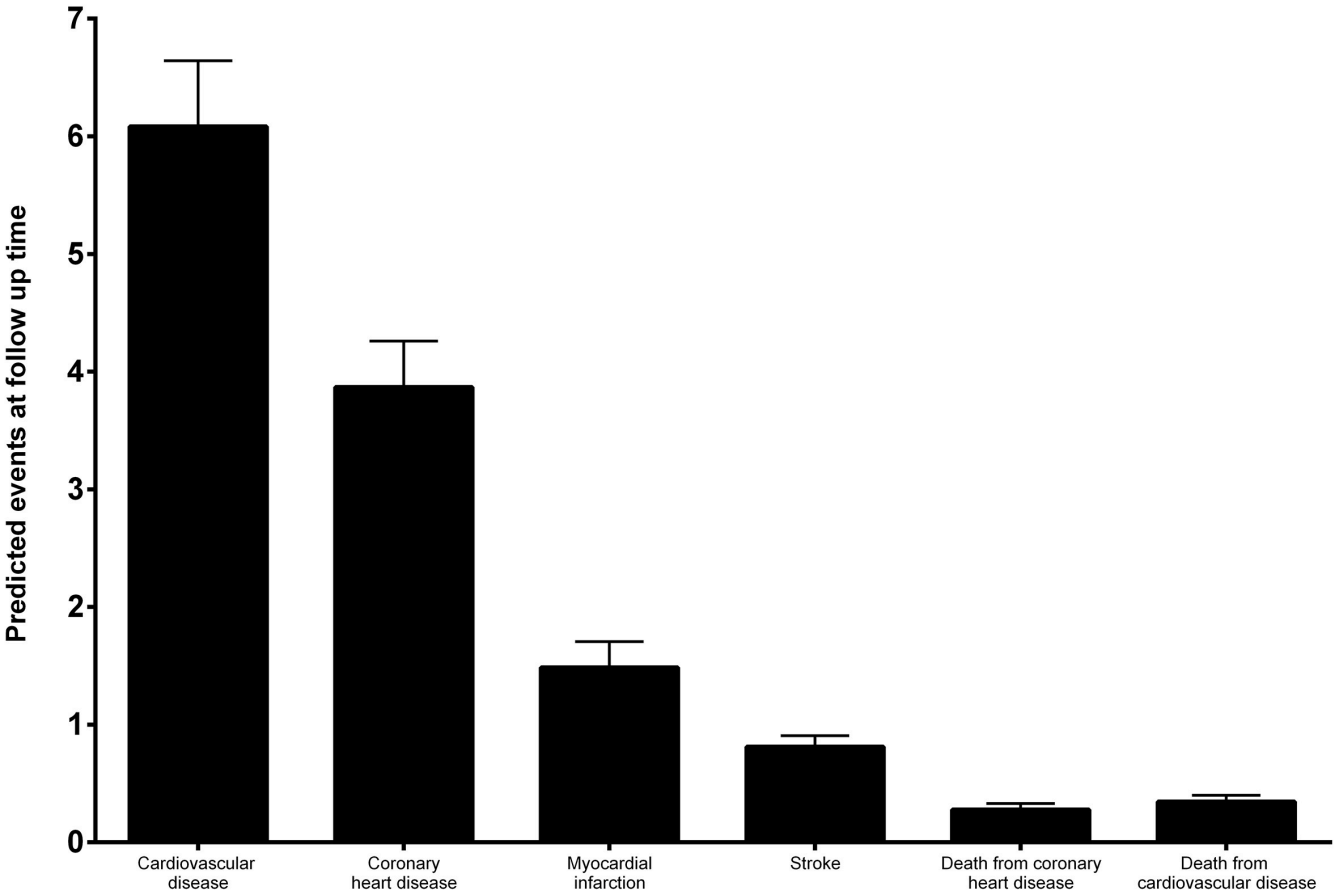
Estimation of the predicted number of CV events during follow-up-time by applying the Framingham Heart Study (2008) score to baseline characteristics of patients with a minimum follow-up of 4 years.

For the subset of patients with more than 4 years of surgery the number of predicted CV events was calculated by adjusting the Framingham Equation to the follow-up time of each individual. Based on the CV risk estimation, 6.08 ± 0.56 CVD events, 3.87 ± 0.39 CHD events, 1.49 ± 0.22 MIs, 0.81 ± 0.09 strokes, 0.28 ± 0.05 deaths from CHD and 0.35 ± 0.05 deaths from CVD were predicted to occur in this subset of patients during the elapsed follow-up time (Figure 4).

No CV events were adjudicated during follow-up time.

## Discussion

Obesity is often associated with a high CV risk that weight loss can mitigate (8, 19). Bariatric surgery induces substantive and sustained weight reduction, in addition to improving several CV risk factors and decreasing CV events and mortality (17, 20, 21). RYGB is a bariatric surgery procedure widely performed for obesity treatment with a well-established effectiveness profile in inducing weight loss in addition to the ability to prevent or even to revert obesity related disorders, such as T2D and CV diseases (12, 22).

In this study we sought to evaluate the CV outcomes associated with weight loss achieved through RYGB surgery by comparing the predicted vs. occurred CV events in a cohort of patients attended in a single academic public center.

In this patient cohort, RYBG surgery induced a significant and sustained weight loss. The maximum weight reduction was achieved 2 years after surgery with a %EBMIL of 76.84%. At that same time point, the estimated 10-year CV risk also decreased significantly when compared to the estimated 10-year CV risk at baseline before the surgical procedure.

Moreover, CV risk reduction was significantly higher in patients with T2D diagnosed prior to surgery. In fact, the 10-year CV risk reductions with greater magnitude were observed for the risks of CHD, MI and CVD, outdoing the CV risk reductions experienced by non-diabetic patients. The sole exception was for the risk of stroke, which was not significantly altered after the intervention, corroborating earlier data from the SOS study, since weight loss after bariatric surgery has not shown to influence the incidence of stroke (23). Remarkably, this observation is quite consistent with other previous findings including those of large trials to assess the cardiovascular outcomes associated with glucose lowering drugs, such as the EMPA-REG trial, which has demonstrated that empaglifozin has several CV benefits in T2D patients and yet it failed to reduce the risk of stroke (24). Furthermore, weight loss elicited by bariatric surgery was proven to be particularly effective in decreasing the CV risk, as well as preventing CV events and mortality in individuals with high insulin and/or glucose levels at baseline (13, 23). Therefore, our data upholds the previous findings further supporting that T2D obese patients submitted to bariatric surgery may have additional CV benefits, besides the well-known glycemic improvement or even T2D clinical remission (17, 23). Subjects with diabetes are known for carrying a disproportionately higher risk of CV disease when compared to non-diabetic subjects. In fact, a significant proportion of T2D patients experience clinical remission of the disease after several bariatric surgical interventions, such as RYGB, along with a significant improvement of several independent CV risk factors, including blood glucose levels, blood pressure and lipid profile, which synergistically result in an overall decrease of the estimated CV risk (14). Moreover, the weight loss profile of patients with T2D diagnosed prior to RYGB surgery was no different from non-diabetic patients, reinforcing the effectiveness of this weight reducing intervention in T2D patients and the likely dissociation of the observed effects in what concerns body weight and CV risk factors.

Age is one of the most important determinants of CV health (25, 26). In earlier times, several national and international guidance's recommended that weight loss surgical interventions should be restricted to patients under 60 years old based on the concerns of age-related increased surgical risks (27). Nonetheless, as the amount of clinical evidence enlarged, it became recognizable that the surgical outcomes of bariatric surgery, including morbidity and mortality rates, in older patients were not significantly different than those observed in younger ones (28–31). Thus, the number of bariatric procedures performed in older patients has increased over the past decades (32, 33). Our data not only reinforces that weight loss achieved after RYGB in patients over 50 years old are not inferior to the observed in younger patients, but also suggests that the CV benefits in this patient group as compared to the younger ones are also more likely to be superior. This difference persists even after the exclusion of T2D patients from analysis, as the decrease in CV risk was still significantly higher in patients older than 50 years, further suggesting that age could be an additional factor to retrieve benefits from surgery.

The fact that our sample size is relatively small when compared to nationwide epidemiologic studies such as the SOS Study, is in no doubt the major limitation of this current study. However, given that our data refers to a single center population, with similar background demographic and clinical features, submitted to a single bariatric surgery technical procedure by the same multidisciplinary team and delivered the same standards of patient care, allowed the retrieval of robust data information for statistical analysis, and thus valid conclusions can be still be ascertained. Nevertheless, the fact that this was a retrospective review of patient records instead of a prospective study designed to assess CV outcomes with formal event adjudication, implies that the level of evidence retrieved from this study is not as high as it would be if derived from a clinical trial. Besides that, there was a 17.5% loss to follow-up rate of subjects that did not have a minimum of 4 years of follow-up after surgery or a minimum of one observation per year during the follow-up time, although, no significant differences in baseline characteristics between participants with long-term follow-up and drop outs were noticed.

Additionally, our data based on CV risk estimated before RYGB surgery predicted that 6 CVD events would have occurred during the elicited follow-up time if patients had not been intervened. However, there were no CV events adjudicated during the same time period. Overall, there was a significant difference between the estimated CV risk and the actual incidence of CV events observed, while RYGB surgery may have prevented 6 CV events in this patient population.

## Conclusion

Weight loss attained trough RYGB surgery was associated with a substantial CV risk reduction and prevented the occurrence of CV events. In addition, diabetic and elder patients experience a superior CV risk reduction and may have additional CV benefits after bariatric surgery, despite depicting similar weight loss and risk factors improvement.

## Author contributions

PP, TM, MG, SP, and MM planned and designed the study. PP, MG, and MN conducted data acquisition. TM performed the statistical analysis. PP, TM, and MM participated in analysis and interpretation of data. PP and MM wrote the manuscript; PP, TM, MG, SP, MN, and MM revised the manuscript. All authors approved the submitted version.

### Conflict of interest statement

The authors declare that the research was conducted in the absence of any commercial or financial relationships that could be construed as a potential conflict of interest.
