# Supplementary material for: Diabetic and Elder Patients Experience Superior Cardiovascular Benefits After Gastric Bypass Induced Weight Loss
Source: Front Endocrinol (Lausanne). 2018 Nov 28;9:718. doi: 10.3389/fendo.2018.00718 (PMC6279895; doi:10.3389/fendo.2018.00718)
Supplement: Supplementary file 1 [file Table_1.DOCX]

| **Table S1.** Comparison of clinical and biochemical features at baseline and 2 years after surgery according to the patients age at the time of RYGB surgery after excluding T2D patients. | | | | | | | | | | | | | | | | | | | | | | | |
| --- | --- | --- | --- | --- | --- | --- | --- | --- | --- | --- | --- | --- | --- | --- | --- | --- | --- | --- | --- | --- | --- | --- | --- |
|  | Age under 50 y | | | | | | |  | Age over 50 y | | | | | | |  | Change from baseline at 2 y | | | | | | |
|  | Baseline | | | 2 y after surgery | | | p |  | Baseline | | | 2 y after surgery | | | p |  | Under 50 y | | | Over 50 y | | | p |
| **Age (years)** | 36.9 | ± | 0.5 |  |  |  |  |  | 55.3 | ± | 0.5 |  |  |  |  |  |  |  |  |  |  |  |  |
| **Male/Female** | 13 |  | 7.6% |  |  |  |  |  | 5 |  | 10.0% |  |  |  |  |  |  |  |  |  |  |  |  |
| **BMI (Kg/m2)** | 43.9 | ± | 0.4 | 29.6 | ± | 0.3 | <0.001 |  | 44.1 | ± | 0.9 | 30.2 | ± | 0.6 | <0.001 |  | -14.3 | ± | 0.3 | -13.9 | ± | 0.6 | 0.33 |
| **Fasting glucose (mg/dL)** | 99 | ± | 2 | 85 | ± | 1 | <0.001 |  | 111 | ± | 5 | 88 | ± | 2 | <0.001 |  | -14 | ± | 3 | -23 | ± | 4 | 0.01 |
| **Total Cholesterol (mg/dL)** | 193 | ± | 3 | 175 | ± | 2 | <0.001 |  | 200 | ± | 5 | 178 | ± | 4 | <0.001 |  | -18 | ± | 3 | -23 | ± | 5 | 0.55 |
| **Triglycerides (mg/dL)** | 131 | ± | 5 | 82 | ± | 3 | <0.001 |  | 129 | ± | 7 | 86 | ± | 4 | <0.001 |  | -48 | ± | 5 | -43 | ± | 7 | 0.77 |
| **LDL (mg/dL)** | 120 | ± | 3 | 102 | ± | 1.2 | <0.001 |  | 129 | ± | 4 | 102 | ± | 4 | <0.001 |  | -19 | ± | 3 | -27 | ± | 5 | 0.22 |
| **HDL (mg/dL)** | 46 | ± | 1 | 57 | ± | 1 | <0.001 |  | 45 | ± | 1 | 58 | ± | 2 | <0.001 |  | 10 | ± | 1 | 13 | ± | 2 | 0.22 |
| **Systolic BP (mm Hg)** | 136 | ± | 1 | 131 | ± | 1 | 0.01 |  | 141 | ± | 3 | 142 | ± | 3 | 0.72 |  | -5 | ± | 1 | -1 | ± | 4 | 0.06 |
| **Diastolic BP (mm Hg)** | 80 | ± | 1 | 78 | ± | 1 | 0.02 |  | 82 | ± | 2 | 82 | ± | 2 | 0.83 |  | -3 | ± | 1 | 0 | ± | 3 | 0.53 |
