# Supplementary figures and images for: Diabetic and Elder Patients Experience Superior Cardiovascular Benefits After Gastric Bypass Induced Weight Loss
Source: Front Endocrinol (Lausanne). 2018 Nov 28;9:718. doi: 10.3389/fendo.2018.00718 (PMC6279895; doi:10.3389/fendo.2018.00718)

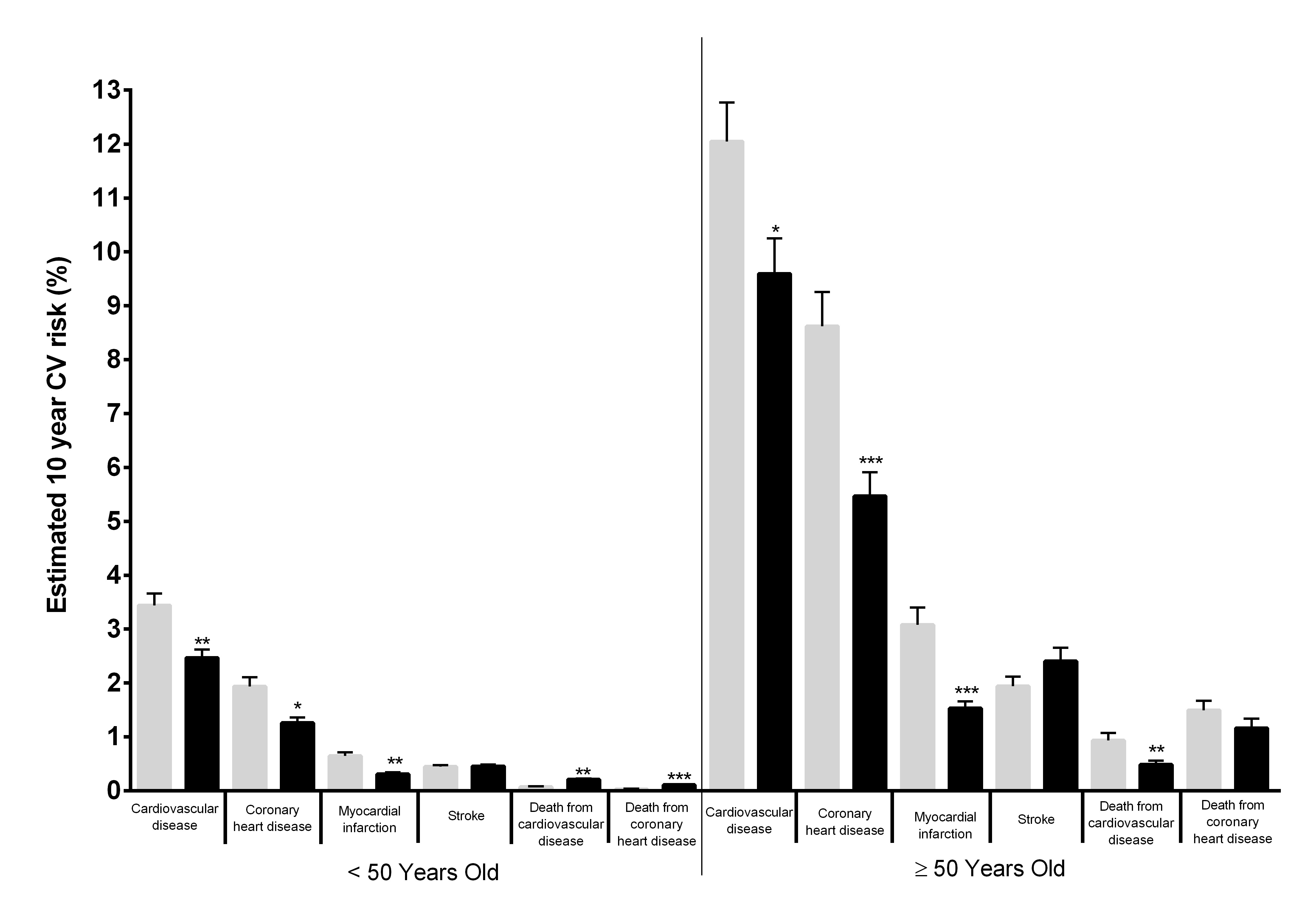

Supplement: Supplementary file 2 [file Image_1.JPEG]
